# A scalable and memory-efficient algorithm for *de novo* transcriptome assembly of non-model organisms

**DOI:** 10.1186/s12864-017-3735-1

**Published:** 2017-05-24

**Authors:** Sing-Hoi Sze, Meaghan L. Pimsler, Jeffery K. Tomberlin, Corbin D. Jones, Aaron M. Tarone

**Affiliations:** 1 Department of Computer Science and Engineering, Texas A&M University, College Station, 77843 TX, USA; 2 Department of Biochemistry & Biophysics, Texas A&M University, College Station, 77843 TX, USA; 3 Department of Entomology, Texas A&M University, College Station, 77843 TX, USA; 4 Department of Biology, University of North Carolina at Chapel Hill, Chapel Hill, NC 27599 USA

**Keywords:** RNA-Seq, Transcriptome assembly, Alternative splicing, Gene expression

## Abstract

**Background:**

With increased availability of *de novo* assembly algorithms, it is feasible to study entire transcriptomes of non-model organisms. While algorithms are available that are specifically designed for performing transcriptome assembly from high-throughput sequencing data, they are very memory-intensive, limiting their applications to small data sets with few libraries.

**Results:**

We develop a transcriptome assembly algorithm that recovers alternatively spliced isoforms and expression levels while utilizing as many RNA-Seq libraries as possible that contain hundreds of gigabases of data. New techniques are developed so that computations can be performed on a computing cluster with moderate amount of physical memory.

**Conclusions:**

Our strategy minimizes memory consumption while simultaneously obtaining comparable or improved accuracy over existing algorithms. It provides support for incremental updates of assemblies when new libraries become available.

## Background

As the advance of high-throughput sequencing makes it possible to sequence billions of bases in a single experiment, this shift in the availability of genomic data allows researchers to focus on biological questions in non-model organisms. With the increased availability of *de novo* assembly algorithms that are designed specifically for assembling millions of short reads [1–8], it becomes possible to study entire genomes or transcriptomes by investigating the assembled sequences.

To obtain a transcriptome, many RNA-Seq libraries are constructed under different experimental conditions or developmental stages, with each library corresponding to one sequencing run of a biological sample. It is preferable to utilize as many libraries as possible to construct one single assembly for each species, as the large amount of data enables simultaneous expression analysis and an increase in coverage support of transcripts that may not be highly expressed under some conditions. In order to obtain the best transcriptomic profile for a given species, there is a need to integrate large amount of accumulated data together from disparate projects and create updated transcriptome assemblies as new data become available. This creates a significant challenge for *de novo* assembly algorithms, since computational resources are often limited in individual labs while the computational time and memory requirement increase rapidly as the number of reads increases.

These computational challenges have motivated the development of algorithms that are specifically designed for performing transcriptome assembly. While algorithms such as Oases [9] and Trinity [8] aim to extract as much information as possible, they are very memory-intensive, limiting their applications to small data sets with few libraries that biologists produce during experiments. Algorithms such as SOAPdenovo-Trans [10] and Trans-ABySS [11] have high memory requirements for large data sets.

To address these difficulties, our goal is to develop transcriptome assembly algorithms that recover alternatively spliced isoforms while utilizing as many RNA-Seq libraries as possible that contain hundreds of gigabases of data. We subdivide the computations into two stages, in which the first stage collects information from each library independently and in parallel, and the second stage merges these results together while minimizing needed computations. To reduce memory consumption so that computations can be performed on a computing cluster with moderate amount of physical memory, we develop new techniques to enumerate *k*-mer frequencies in the first stage. We impose appropriate cutoffs in the second stage in order to obtain comparable or improved accuracy over existing algorithms. This strategy supports incremental updates of assemblies when new libraries become available since only the second stage needs to be rerun.

We evaluate the performance of our algorithm by constructing transcriptome assemblies using publicly available libraries from model organisms, and comparing our assemblies to the ones obtained from SOAPdenovo-Trans, Trans-ABySS, Oases and Trinity. We evaluate our performance on non-model organisms both by obtaining publicly available libraries from the naked mole rat *Heterocephalus glaber* and by constructing new RNA-Seq libraries for the blow fly *Chrysomya rufifacies*.

## Methods

### De Bruijn graph

Given a set of reads and a parameter *k*, a de Bruijn graph is defined by taking each *k*-mer that appears within the reads as a vertex. Two *k*-mers *s*_1_ *s*_2_⋯*s*_*k*_ and *s*_2_⋯*s*_*k*_ *s*_*k*+1_ are connected by a directed edge if the (*k*+1)-mer *s*_1_ *s*_2_⋯*s*_*k*_ *s*_*k*+1_ appears in the reads and the (*k*−1)-suffix of the first *k*-mer is the same as the (*k*−1)-prefix of the second *k*-mer, where *s*_1_ and *s*_*k*+1_ can be arbitrary letters. By linking together the same *k*-mer that appears in different reads, the de Bruijn graph can be used to implicitly assemble these reads [12, 13]. Since the size of the de Bruijn graph depends on the number of distinct *k*-mers from the reads that is often much smaller than the total size of reads, this strategy is especially suitable for assembling high-throughput sequencing data [2, 3, 5–7].

### Independent computation of *k*-mer frequencies

While most existing short read assembly algorithms use hashing techniques [5, 8, 14] or suffix arrays [15] to enumerate *k*-mer frequencies, the memory requirement per *k*-mer can be high with large multiplicative constants. While techniques such as sparse hashing (http://code.google.com/p/google-sparsehash) or entropy-based compression [16] can be used to reduce the memory overhead per *k*-mer, there is a need to handle collisions and the memory requirement can still be high. Recently, two algorithms DSK [17] and KMC [18] were developed based on disk-based partitioning of the *k*-mer space, which allow the user to specify a memory consumption limit.

**Fig. 1:**
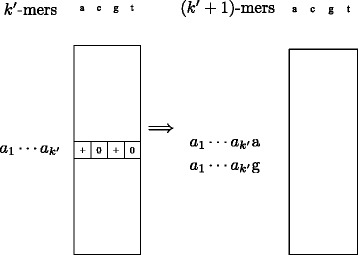
Illustration of the iterative algorithm to enumerate *k*-mer frequencies. For the *k* ^′^-mer $\protect \phantom {\dot {i}\!}a_{1}\cdots a_{k^{\prime }}$, its two frequency slots with zero counts for nucleotides c and t are removed to obtain (*k* ^′^+1)-mers $\protect \phantom {\dot {i}\!}a_{1}\cdots a_{k^{\prime }}$a and $\protect \phantom {\dot {i}\!}a_{1}\cdots a_{k^{\prime }}$g

We consider the following iterative algorithm to enumerate *k*-mer frequencies independently for each library (see Fig. 1). At the start of each iteration, we assume that a list of all *k* ^′^-mers that appear in the library in either the forward or the reverse complementary direction are given in sorted order for *k* ^′^<*k*. This list can be represented by encoding each nucleotide by two bits and using a 64-bit or 128-bit integer to encode each *k* ^′^-mer. Suppose that there are *n* such *k* ^′^-mers. We create an array of size 4*n* that contains four slots for each *k* ^′^-mer. We use it to count the number of each (*k* ^′^+1)-mer that appears in the library by using binary search to find the location of its *k* ^′^-prefix within the array and updating one of the four slots that corresponds to its last nucleotide. At the end of the iteration, we remove slots with zero counts to obtain a list of all (*k* ^′^+1)-mers.

To make sure that each edge in the de Bruijn graph corresponds to a (*k*+1)-mer that appears in the library, we repeat this procedure until the frequencies of (*k*+1)-mers are obtained and store all *k*-mer frequencies along with edge information. To reduce computational time, we start the process with a moderate value of *k* ^′^ (between 10 to 15) by assuming that all *k* ^′^-mers appear in the library. One advantage of this procedure is that the memory requirement per *k*-mer is low with a multiplicative constant of four. When multiple assemblies with different values of *k* are needed, frequencies with smaller values of *k* for one assembly can be used to obtain frequencies with larger values of *k* for another assembly. This significantly reduces the computational time over all values of *k*.

### Construction of de Bruijn graph

Given a list of all *k*-mer frequencies in sorted order for each library, we combine these lists by performing a merge sort and adding the corresponding frequencies for each *k*-mer. We exclude a *k*-mer from the de Bruijn graph if its frequency is less than a given *k*-mer coverage cutoff *c*. This strategy is different from the one employed by other short read assembly algorithms such as Velvet [5] or ABySS [6], which apply the cutoff after constructing the de Bruijn graph and removing redundant paths that are of less support. Since our results show that there is a need to increase the values of *k* and *c* to obtain good performance as the size of the data set increases, our strategy keeps the size of the de Bruijn graph manageable. Edges in the de Bruijn graph are constructed by linking together adjacent *k*-mers through binary search. After the initial construction of the de Bruijn graph, each linear path that contains a maximal succession of vertices with no branches is collapsed into a single node. While the entire merging process needs to be run sequentially for each given setting of *k* and *c*, different settings of *k* and *c* can be run in parallel.

### Construction of splicing graphs

To simplify the de Bruijn graph, we remove short tips that may correspond to sequencing errors by iteratively removing end nodes with sequence length less than 2*k*. We follow the strategy in [19] to obtain splicing graphs from the de Bruijn graph so that each splicing graph mostly represents alternatively spliced variants of only one gene, with new strategies to handle paired-end reads. Note that this strategy is different from algorithms that construct a set of predicted transcripts from the de Bruijn graph [8–11].

In order to remove obvious SNPs that create branches in the graph, we search for split-then-merge branching structures in which all the branches from a node merge immediately into a single node and the sequences associated with each branch are of the same length with very few mismatches. Successive split-then-merge structures are merged into a single node.

For each paired-end read, we identify the node *u* in the collapsed de Bruijn graph in which the last *k*-mer of the forward read resides and the node *v* in which the first *k*-mer of the reverse read resides, and increase the frequency count of the paired edge *u*→*v* by one. We repeat this procedure over all paired-end reads and retain all paired edges that have frequency counts above a given cutoff *c*_2_ that is proportional to the total number of bases in the data set. The resulting de Bruijn graph contains two types of edges, including normal edges and paired edges.

Our results show that there is always a big tangle in the de Bruijn graph that contains a large number of nodes within a single connected component, while most of the other tangles are much smaller. In order to address these tangles, we decompose each connected component into strongly connected components, in which each strongly connected component is either just a single edge or a maximal subgraph with each node reachable from all other nodes. The regions within a strongly connected component that are not just a single edge represent the complicated regions that always contain a cycle, while the other regions represent the simpler regions in which each connected region is likely to belong to the same gene. With the assumption that the reads are not strand-specific, it is also possible to have forward-backward tangles in which a connected component contains both a forward node and its corresponding backward node in the reverse complementary direction. Both of these structures can be identified by using depth-first search with time complexity that is linear in the size of the graph.

We extract the strongly connected components that are not just a single edge and all the forward-backward tangles. We ignore the junction information within these subgraphs, and treat each node as an individual splicing graph that consists only of a single node. We remove these nodes along with their adjacent edges, and extract each connected component in the remaining graph as a splicing graph that does not contain cycles. We remove the overlapping sequence fragments within the nodes that arise according to the definition of a de Bruijn graph, and make the junction locations precise in obvious cases when a node does not have multiple incoming edges and multiple outgoing edges at the same time. We retain one of the two possible orientations for each splicing graph. Only splicing graphs with length (in nucleotides) of the longest path of at least 100 are retained.

In order to study expression of nodes in a splicing graph, we incorporate the *de novo* expression measure of number of reads per kilobase of node per million reads (RPKM) developed in [19]. This measure is similar to the number of reads per kilobase of exon per million mapped reads used by [20] and [21], except that reads that appear in the assembly are used instead of mapped reads, and each node in a splicing graph is evaluated instead of each exon, with each read that contains a *k*-mer within a node contributing to that node. Within each node, a RPKM estimate is computed independently for each library. Alternatively, measures similar to transcripts per million (TPM) [22] can be used, which are more comparable across libraries.

In order to make the results directly applicable to downstream analysis, we represent each assembly in an annotated FASTA format, in which each splicing graph is given as a collection of nodes, with connecting normal and paired edges and RPKM values for each library embedded within the name of each node. Since it is possible to have empty nodes that do not contain any nucleotide after adjustment of junction locations, RPKM values are computed before junction adjustment to reflect the original coverage values across a branch. Such empty nodes correspond to additional isoforms that skip nodes (e.g., exons) within a branch.

## Results and discussion

### Model organisms

To compare the performance of our algorithm ASplice to other algorithms, we extracted reads from publicly available RNA-Seq libraries in model organisms *Schizosaccharomyces pombe*, *Arabidopsis thaliana* and *Drosophila melanogaster* (see Table 1). We trimmed each read by removing all positions including and after the first position that has a quality score of less than 15. We applied our algorithm to obtain a de Bruijn graph for a given *k*-mer length and a given *k*-mer coverage cutoff *c*. We compare the performance of our algorithm ASplice to SOAPdenovo-Trans and Trans-ABySS on machines with 32 GB physical memory (except for *D. melanogaster*, in which there is not sufficient memory to run SOAPdenovo-Trans). Nucleotide BLAST search is applied to the transcriptome of the same organism to evaluate the performance. Since each algorithm returns different structures, note that the results are not completely comparable.

**Table 1:** Data sets used in the evaluation of transcriptome assembly, with organism denoting the organism, type denoting whether the organism is model or non-model, libraries denoting the total number of libraries, size denoting the total number of bases in all the reads after quality trimming, reference denoting the publication that describes the libraries, and tick marks within assembly on 32 GB machines denoting the algorithms that can be used for assembly on machines with 32 GB physical memory

					Assembly on 32 GB machines		
Organism	Type	Libraries	Size	Reference	SOAPdenovo-Trans	Trans-ABySS	ASplice
*S. pombe*	Model	32	16.9 G	[8]	*√*	*√*	*√*
*A. thaliana*	Model	5	16.1 G	[29]	*√*	*√*	*√*
*D. melanogaster*	Model	245	158 G	[30]		*√*	*√*
*H. glaber*	Non-model	13	60.5 G	[31]		*√*	*√*
*C. rufifacies*	Non-model	66	590 G	New data			*√*

Table 2 shows that while there were performance tradeoffs among different values of *k* and the *k*-mer coverage cutoff *c*, SOAPdenovo-Trans and Trans-ABySS generally recovered longer transcript structures and more genes in the transcriptome (except for *S. pombe*, in which ASplice recovered more genes).

**Table 2:** Comparisons of transcriptome assemblies of SOAPdenovo-Trans, Trans-ABySS and ASplice in model organisms over different values of *k* and *k*-mer coverage cutoff *c*

*S. pom*	SOAPdenovo-Trans					Trans-ABySS					ASplice				
			total	unique	mem			total	unique	mem	splicing		total	unique	mem
*k*_*c*	locus	N50	hits	hits	(GB)	trans	N50	hits	hits	(GB)	graphs	N50	hits	hits	(GB)
25_10	3267	4455	7343	4230	10	21215	2854	28376	4400	3	5859	3032	5271	4650	9
25_20	3393	3795	7153	4341	10	14193	2880	19541	4284	3	5231	2723	4941	4579	9
25_50	3747	3025	8174	4513	10	8748	2317	10686	4370	3	5163	2210	5002	4580	9
31_10	3366	4164	8481	4342	10	20569	2977	33192	4323	4	5580	3005	5148	4611	9
31_20	3470	3590	7864	4418	10	13701	3045	22303	4158	4	5076	2625	4956	4565	9
31_50	3891	2788	8783	4576	10	7972	2421	10259	4284	4	5103	2088	5080	4620	9
*A. tha*	SOAPdenovo-Trans					Trans-ABySS					ASplice				
			total	unique	mem			total	unique	mem	splicing		total	unique	mem
*k*_*c*	locus	N50	hits	hits	(GB)	trans	N50	hits	hits	(GB)	graphs	N50	hits	hits	(GB)
25_10	18980	1705	80644	21614	17	348069	460	383663	21715	7	103450	344	92605	21487	10
25_20	16327	1622	71684	20239	17	159952	772	139875	20213	7	71419	561	66645	20033	9
25_50	13384	1472	53209	17788	17	66058	948	70136	17544	7	43407	778	42396	17535	9
31_10	19604	1700	74854	20952	17	350165	578	525359	21422	7	92665	444	88408	21288	10
31_20	16882	1605	62748	19516	17	141642	948	181322	19838	7	58877	760	56869	19691	9
31_50	13660	1438	42763	16990	17	54189	1083	75863	17050	7	35448	973	34906	17030	9
*D. mel*	Trans-ABySS								ASplice						
			total		unique		mem		splicing			total	unique		mem
*k*_*c*	trans	N50	hits		hits		(GB)		graphs		N50	hits	hits		(GB)
25_10	135048	1192	179499		12989		27		99930		728	51854	12322		11
25_20	83303	1693	102591		12848		27		60662		1328	35402	12245		10
25_50	47341	2082	58523		12453		27		36093		1874	26130	11921		9
31_10	113805	1547	225887		13025		30		77439		1203	45278	12453		11
31_20	70061	2029	124050		12787		30		45645		1952	28616	12259		10
31_50	41210	2296	64736		12337		30		32593		2085	24093	11861		9

The predicted units are locus for SOAPdenovo-Trans that is represented as a splicing graph containing nodes and edges, transcript (trans) for Trans-ABySS that is a linear concatenation of constituent nodes, and splicing graph for ASplice. For SOAPdenovo-Trans and ASplice, N50 denotes the N50 value of the length (in nucleotides) of the longest path in each splicing graph. For Trans-ABySS, N50 denotes the N50 value of the length of a predicted transcript, and only predicted transcripts of length at least 100 are retained. Total hits denotes the total number of hits from nucleotide BLAST search of nodes to the transcriptome of the same organism. Isoforms are considered to be the same gene. Only the top hit with *E*-value below 10^−7^ is considered. Hits from nodes within the same predicted unit to the same gene are counted only once. Unique hits denotes the number of unique hits to different genes. Mem (GB) denotes the physical memory requirement in gigabytes over all stages of each algorithm

**Fig. 2:**
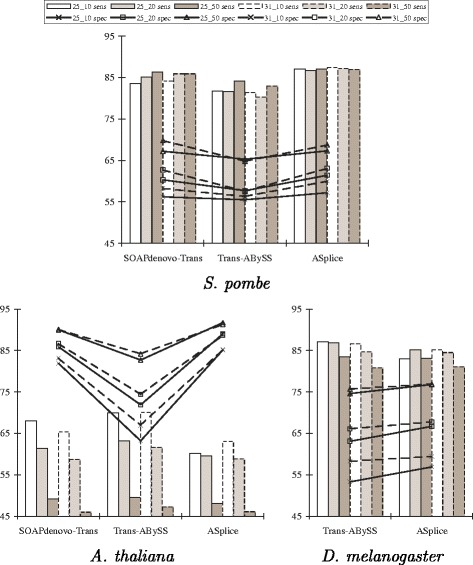
Sensitivity and specificity comparisons of SOAPdenovo-Trans, Trans-ABySS and ASplice with respect to mRNA BLAST results in model organisms over different values of *k* and *k*-mer coverage cutoff *c* (represented by *k*_*c*). Sensitivity (sens; marked by bars) is defined to be the percentage of nucleotide positions in the transcriptome that are recovered through the top BLAST alignments from each node in the assembly. Specificity (spec; marked by lines) is defined to be the percentage of predicted positions that are included in the top BLAST alignments from each node of the assembly

Figure 2 shows that ASplice had higher specificity with respect to the percentage of predicted positions that are included in the top BLAST alignments. ASplice also often had comparable sensitivity with respect to the percentage of nucleotide positions in the transcriptome that are recovered through the top BLAST alignments, especially for larger values of the *k*-mer coverage cutoff *c* when the assembly conditions are more stringent, and had higher sensitivity for *S. pombe*.

**Fig. 3:**
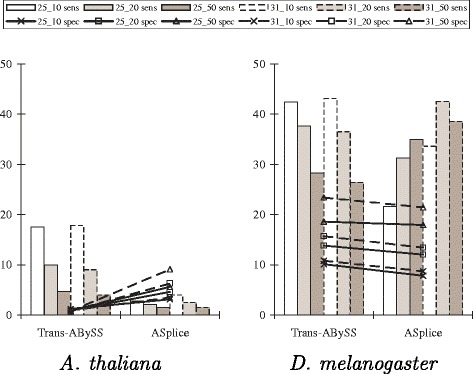
Sensitivity and specificity comparisons of Trans-ABySS and ASplice with respect to alternative splicing junctions in model organisms over different values of *k* and *k*-mer coverage cutoff *c* (represented by *k*_*c*). Sensitivity (sens; marked by bars) is defined to be the percentage of junctions in the gene transcripts that appear in the assembly. Specificity (spec; marked by lines) is defined to be the percentage of junctions in the assembly that appear in the gene transcripts. Junctions in the gene transcripts are defined by concatenating the two sequences of length *k* that are immediately to the left and immediately to the right of all locations with alternative splicing that are derived from annotated positions of the gene transcripts to obtain a sequence of length 2*k*. Junctions in the assembly are defined by concatenating the two non-overlapping *k*-mers at the beginning and ending nodes of an edge to obtain a sequence of length 2*k*. Up to three mismatches are allowed when looking for occurrences of these sequences that span across a junction. Notations are the same as in Fig. 2

Figure 3 further shows that, with respect to alternative splicing junctions that are derived from the splicing graphs and annotated positions of the gene transcripts, ASplice was often more conservative, and could have higher sensitivity when the assembly conditions are more stringent (no comparisons were made to SOAPdenovo-Trans since its assemblies often contain many gap positions around junctions, making comparisons difficult). The poor performance with respect to alternative splicing junctions in *A. thaliana* is due to the relatively small size of the data set.

**Fig. 4:**
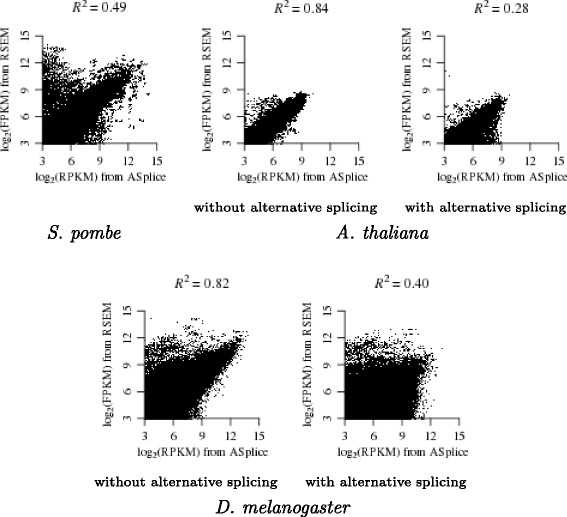
Regression in log scale of the *de novo* RPKM values from the 25_10 assembly (with *k*=25 and *k*-mer coverage cutoff *c*=10) of ASplice against the FPKM values from RSEM over all libraries. The gene transcript that corresponds to each node from ASplice is identified through a bi-directional nucleotide BLAST alignment of length at least 100 to the transcriptome of the same organism. Nodes that have no correspondences are ignored. For *A. thaliana* and *D. melanogaster*, regressions are performed separately on gene transcripts without alternative splicing and on gene transcripts with alternative splicing

Figure 4 shows that our *de novo* expression estimates were highly correlated to the ones obtained from applying RSEM [22] to map the reads in each library to the transcriptome of the same organism, with 0.49≤*R* ^2^≤0.84 for gene transcripts without alternative splicing and 0.28≤*R* ^2^≤0.40 for gene transcripts with alternative splicing, where *R* ^2^ denotes the coefficient of determination that measures how well the data fit a regression line. The lower *R* ^2^ in the latter case is probably due to higher assembly difficulties.

In terms of memory requirement, Table 2 shows that while SOAPdenovo-Trans and Trans-ABySS had large increases as the size of the data set increases (compare to Table 1), ASplice had a large fixed memory overhead for all data sets with small increases for larger data sets. Since each library can be further subdivided into multiple parts to reduce memory requirement during the parallel stage of ASplice, the maximum memory consumption is obtained during the sequential stage, in which the main structure to store is the de Bruijn graph after the *k*-mer coverage cutoff *c* is applied.

### Non-model *Heterocephalus glaber*

We also assess the performance of our algorithm in the non-model naked mole rat *Heterocephalus glaber* (there is not sufficient memory to run SOAPdenovo-Trans on machines with 32 GB physical memory for this data set). Table 3 shows that large values of the *k*-mer coverage cutoff *c* were needed to obtain reasonable assemblies due to the large size of the data set (see also Table 1). Similar trends were observed as before when translated BLAST search to the rat *Rattus norvegicus* is applied, with ASplice recovering more genes when the assembly conditions are more stringent.

**Table 3:** Comparisons of transcriptome assemblies of Trans-ABySS and ASplice in the naked mole rat *H. glaber* over different values of *k* and *k*-mer coverage cutoff *c*

*H. gla*	Trans-ABySS						ASplice			
			*R. nor*	unique	mem	splicing		*R. nor*	unique	mem
*k*_*c*	trans	N50	hits	hits	(GB)	graphs	N50	hits	hits	(GB)
25_50	97640	970	40391	13592	19	110379	533	35671	13056	9
25_100	62495	714	34433	11758	19	71037	445	30442	11558	9
25_200	37371	527	24940	9059	19	42666	359	21515	9371	9
31_50	91149	987	38346	13415	20	116375	457	38271	12900	9
31_100	59730	695	33864	11510	20	73734	381	31369	11356	9
31_200	35292	503	24404	8703	20	42180	320	21110	9084	9

Notations are the same as in Table 2 except that translated BLAST search (to *R. norvegicus*) is performed instead of nucleotide BLAST search

### Non-model *Chrysomya rufifacies*

We applied our algorithm to assemble the transcriptome of the blow fly *Chrysomya rufifacies* from a set of RNA-Seq libraries that we have constructed, which includes the following developmental stages: embryos, first instar larvae, second instar larvae, predator and non-predator third instar larvae, early pupae, mid pupae, late pupae, thelygenic and arrhenogenic adult females, and adult males. There are totally 66 libraries with 6.8 G reads and average read length 86 after quality trimming.

The blow fly *C. rufifacies* has monogenic sex determination in which a female either produces only female offspring (thelygenic) or produces only male offspring (arrhenogenic) based on the genotype [23], which is a distinct mechanism among flies. Sex determination in flies is typically achieved (in part) by alternative splicing, in which sex-specific isoforms of genes like *doublesex* and *transformer* lead to female or male development [24, 25]. Within the genus, there is also a human-associated male-eye phenotype that is hypothesized to have evolved multiple times in concert with human civilization [26]. Genomic tools for this blow fly enable the study of the evolution of sex determination and co-evolution with humans.

Since there is not sufficient memory to run either SOAPdenovo-Trans or Trans-ABySS on machines with 32 GB physical memory for this large data set with 590 G bases, we only run ASplice, which allows computations to be performed on a computing cluster due to its low memory requirements. We considered larger values of *k* and further subdivided large libraries into multiple parts during the parallel stage. Table 4 shows that the assemblies were of high quality, with long splicing graphs, moderate amount of branches that may represent alternative splicing, and over 60% of the *D. melanogaster* genes recovered. The ratio of the total number of BLAST hits from different splicing graphs to the number of unique BLAST hits to different genes was between 1.5 to 2, indicating a small amount of sequence fragmentation of the same gene into different splicing graphs. There were only a small number of splicing graphs that have BLAST hits to more than one gene, and the maximum number of different genes that have BLAST hits to a splicing graph was small, thus each splicing graph specifies the alternatively spliced variants of one gene in most cases.

**Table 4:** Transcriptome assemblies of ASplice in the blow fly *C. rufifacies* over different values of *k* and *k*-mer coverage cutoff *c*

*C. ruf*	splicing	max		>1-node	max	avg	*D. mel*	unique	>1-hit	max	mem
*k*_*c*	graphs	length	N50	graphs	nodes	nodes	hits	hits	graphs	hits	(GB)
31_50	67945	29763	1482	20900	2066	33	17516	9246	1029	15	13
31_100	52099	27855	1651	16790	2786	27	16237	9090	844	38	10
31_200	41872	35802	1717	13318	2648	21	15013	8776	624	68	9
35_50	66381	55047	1600	20465	5299	33	16470	9235	994	59	13
35_100	53120	34347	1686	16529	1398	26	15965	9062	769	14	10
35_200	41421	35802	1769	13159	4076	21	14693	8723	582	76	9

Max length denotes the length (in nucleotides) of the longest path over all splicing graphs. >1-node graphs denotes the number of splicing graphs with non-linear structures. Max nodes denotes the maximum number of nodes in a splicing graph. Avg nodes denotes the average number of nodes in splicing graphs with non-linear structures. >1-hit graphs denotes the number of splicing graphs that have BLAST hits to more than one gene in *D. melanogaster*. Max hits denotes the maximum number of different genes that have BLAST hits to a splicing graph. Other notations are the same as in Table 2 except that translated BLAST search to *D. melanogaster* is performed

**Fig. 5:**
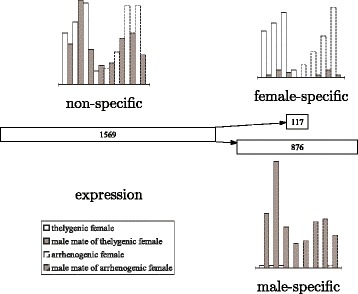
A splicing graph in the 31_200 assembly (with *k*=31 and *k*-mer coverage cutoff *c*=200) of ASplice in the blow fly *C. rufifacies* that is related to the *doublesex* gene in *D. melanogaster*. Each node is represented by a rectangle of width that is proportional to (and labeled by) the length of its sequence. The histogram adjacent to each node gives the RPKM values for each adult library, with each light bar and the dark bar to its right denoting an adult female and its mate (an adult male) respectively. The left half of each histogram (marked by solid bars) denotes thelygenic females and their mates, while the right half of each histogram (marked by dashed bars) denotes arrhenogenic females and their mates

By comparing to the *D. melanogaster* homologs of assembled nodes (see Fig. 5), we found expected alternative splicing in the *doublesex* gene and consistent bias of expression within female-specific (upper right node of length 117) and male-specific (lower right node of length 876) segments and between thelygenic and arrhenogenic females (with generally higher expression within thelygenic females in the upper right node).

### Small *Drosophila melanogaster* libraries

Since Oases and Trinity are very memory-intensive, we assess their performance by extracting reads from a small set of three *D. melanogaster* RNA-Seq libraries in [27] at the sequence read archive [28] that includes the following developmental stages: 2–16 hours embryos (SRR058885), third instar larvae (SRR059066), and mixed pupae (SRR042298). These libraries have 1.8 G bases after quality trimming, and *k* is fixed to 25.

**Fig. 6:**
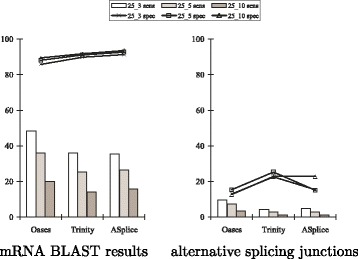
Sensitivity and specificity comparisons of Oases, Trinity and ASplice with respect to mRNA BLAST results and alternative splicing junctions on a small set of libraries from *D. melanogaster* with *k*=25 and over different values of *k*-mer coverage cutoff *c* (represented by *k*_*c*). Notations are the same as in Figs. 2 and 3

Table 5 shows that Oases had the longest assemblies and recovered the largest number of genes. While ASplice had longer assemblies than Trinity, Trinity recovered more genes. Figure 6 shows that ASplice had slightly higher specificity at the expense of lower sensitivity with respect to recovered mRNA positions, and had higher specificity with respect to alternative splicing junctions when the assembly conditions are more stringent. Sensitivity was low for all algorithms due to the small size of the data set. When compared to the results on the large *D. melanogaster* data set, this shows that as many libraries as possible should be utilized to obtain a more complete transcriptome, justifying the use of our scalable algorithm when possible.

**Table 5:** Comparisons of transcriptome assemblies of Oases, Trinity and ASplice on a small *D. melanogaster* data set with *k*=25 and over different values of *k*-mer coverage cutoff *c*

*D. mel*	Oases					Trinity					ASplice				
(small)			total	unique	mem			total	unique	mem	splicing		total	unique	mem
*k*_*c*	locus	N50	hits	hits	(GB)	locus	N50	hits	hits	(GB)	graphs	N50	hits	hits	(GB)
25_3	32277	748	46371	11311	26	50656	264	48079	9987	12	31949	305	30423	9560	9
25_5	21246	880	28366	9930	20	35797	254	34487	8412	12	20533	335	20061	8043	9
25_10	11509	842	15489	7075	18	20281	245	19759	5946	12	11243	338	11149	5722	9

For Oases and Trinity, the predicted unit is locus that contains a set of predicted transcripts, N50 denotes the N50 value of the longest transcript length in a predicted locus, and predicted transcripts of length at least 100 are retained. Other notations are the same as in Table 2

## Conclusions

We have developed an algorithm for *de novo* transcriptome assembly of non-model organisms that utilizes a large amount of RNA-Seq libraries in order to obtain a transcriptome that is as complete as possible, while simultaneously extracting alternative splicing information and expression levels in different libraries. When compared to existing algorithms, our algorithm is more conservative and generally has higher specificity at the expense of lower sensitivity, but is able to utilize larger amount of data to obtain more complete assemblies. As the size of the data set increases, larger values of *k* and usually much larger values of the *k*-mer coverage cutoff *c* are needed to obtain reasonable assemblies.

Since large libraries can be further subdivided into multiple parts during the parallel stage, our algorithm is scalable and the parallel stage can be run on different computing nodes. Since our *k*-mer counting technique requires iterating over every read and performing binary search repeatedly over increasing values of *k*, our algorithm is generally much more computationally intensive than existing algorithms, although our memory requirement is much lower for large data sets. Our strategy is especially suitable when multiple assemblies with different values of *k* are desired, as our algorithm is based on processing iteratively larger values of *k*. Incremental updates of assemblies are easy to perform, as it is only necessary to run the parallel stage on the new libraries before running the sequential stage on all libraries. Such a strategy is especially important to iteratively obtain more complete transcriptome assemblies over time through collaboration across research communities.

For small to medium data sets, it takes a few hours to a few days to complete the parallel stage over all values of *k* as long as the libraries are subdivided into small enough parts. The time to finish the sequential stage ranges from a few hours for a small data set to one to two days for a larger data set. For our largest *C. rufifacies* data set with 590 G bases, it takes a few days to finish the parallel stage over all values of *k* when the libraries are subdivided into parts with about 10 G bases each, with each computing process consuming less than 14 GB memory. For each given setting of *k* and the *k*-mer coverage cutoff *c*, it takes a few days to finish the sequential stage. With values of *c* between 50 and 200 in our largest *C. rufifacies* data set, the memory consumption is less than 14 GB. Thus, our algorithm can assemble large data sets on a computing cluster with moderate resources.
